# The quantal theory of how the immune system discriminates between "self and non-self"

**DOI:** 10.1186/1476-9433-3-3

**Published:** 2004-12-17

**Authors:** Kendall A Smith

**Affiliations:** 1 The Division of Immunology, Department of Medicine, Weill Medical College, Cornell University, New York, New York, United States of America

## Abstract

In the past 50 years, immunologists have accumulated an amazing amount of information as to how the immune system functions. However, one of the most fundamental aspects of immunity, how the immune system discriminates between self vs. non-self, still remains an enigma. Any attempt to explain this most intriguing and fundamental characteristic must account for this decision at the level of the whole immune system, but as well, at the level of the individual cells making up the immune system. Moreover, it must provide for a molecular explanation as to how and why the cells behave as they do. The "Quantal Theory", proposed herein, is based upon the "Clonal Selection Theory", first proposed by Sir McFarland Burnet in 1955, in which he explained the remarkable specificity as well as diversity of recognition of everything foreign in the environment. The "Quantal Theory" is built upon Burnet's premise that after antigen selection of cell clones, a proliferative expansion of the selected cells ensues. Furthermore, it is derived from experiments which indicate that the proliferation of antigen-selected cell clones is determined by a quantal, "*all-or-none*", decision promulgated by a critical number of cellular receptors triggered by the T Cell Growth Factor (TCGF), interleukin 2 (IL2). An extraordinary number of experiments reported especially in the past 20 years, and detailed herein, indicate that the T cell Antigen Receptor (TCR) behaves similarly, and also that there are several critical numbers of triggered TCRs that determine different fates of the T cells. Moreover, the fates of the cells appear ultimately to be determined by the TCR triggering of the IL2 and IL2 receptor (IL2R) genes, which are also expressed in a very quantal fashion. ***The "Quantal Theory" states that the fundamental decisions of the T cell immune system are dependent upon the cells receiving a critical number of triggered TCRs and IL2Rs and that the cells respond in an all-or-none fashion***. The "Quantal Theory" accounts fully for the development of T cells in the thymus, and such fundamental cellular fates as both "positive" and "negative" selection, as well as the decision to differentiate into a "Regulatory T cell" (T-Reg). In the periphery, the "Quantal Theory" accounts for the decision to proliferate or not in response to the presence of an antigen, either non-self or self, or to differentiate into a T-Reg. Since the immune system discriminates between self and non-self antigens by the accumulated number of triggered TCRs and IL2Rs, therapeutic manipulation of the determinants of these quantal decisions should permit new approaches to either enhance or dampen antigen-specific immune responses.

## Introduction

Perhaps one of the most unique and fundamental aspects of the immune system is the clonal nature of the response to the introduction of antigen. The "Clonal Selection Theory" as originally formulated by Burnet stated that the immune system is made up of cells, each of which are only capable of reacting with a single antigenic molecule[1]. Thus, Burnet improved upon the "Natural-selection theory of antibody formation" proposed by Neils Jerne[2], by giving the immune response a cellular basis. Also, Burnet introduced the notion that after antigen selection, the reactive cell clones proliferate, and the resulting expanded population of cells would only then be capable of removing the offending foreign antigen. Consequently, one of the most crucial decisions required of an antigen-selected cell is whether to undergo cell cycle progression. Ultimately this decision determines one of the other most fundamental characteristics of the immune system, the ability to discriminate between self vs. non-self.

At the time that Burnet formulated the concept of clonal selection in the mid 1950s, the identity of the cells comprising the immune system was unknown. Plasma cells had been found to be the source of antibodies[3], but lymphocytes had not yet been identified as the precursors of plasma cells. Moreover, thymic-derived lymphocytes (T cells) and bone marrow-derived lymphocytes (B cells) were not to be discovered for almost two more decades [4-6].

In the intervening 50 years since Burnet formulated his theory, much has been discovered and even more has been proposed to explain how the immune system functions, especially how the discrimination between self vs. non-self is made. Since 1959, several modifications of Burnet's original model have been offered, each of which introduced additional cells in an attempt to explain how the entire system could react with absolutely everything in the environment, but not react with any molecules comprising self [7-15]. However, none of the models proposed thus far have focused on one of the most fundamental aspects of the Clonal Selection Theory as originally formulated by Burnet, i.e. the molecular forces driving the proliferative expansion of the antigen-selected cell clones.

We now know that lymphocytes make up the immune system, and we know the molecular structures of the antigen receptors expressed by both T cells and B cells. Consequently we know that Burnet was largely correct. Each cell expresses a unique antigen receptor that has the capacity to bind only a single antigenic epitope. The exception to this rule is the "allelic inclusion" of two T Cell Receptor (TCR) α-chains, resulting in approximately 30% of α/β-chain bearing T cells as having dual antigenic specificity. As well, it is known that T cells recognize epitopes comprised of short peptides of only a few amino acid residues bound to molecules encoded by the Major Histocompatibility gene Complex (MHC), while B cells recognize both the tertiary surface structure of larger molecules, as well as linear determinants of molecules. Despite this information, it still remains unknown how the immune system discriminates between non-self vs. self-molecules, in that the molecular nature of the T cell Antigen Receptors (TCR) and the B cell Antigen Receptors (BCR) that recognize both self- and non-self- epitopes are identical. Moreover, the molecular natures of self-epitopes and non-self -epitopes are identical. Accordingly, it is even more perplexing as to how the immune system manages this discrimination.

One key to understanding the way in which the immune system operates is the observation that the capacity to recognize and respond to antigen is dependent on the dose of antigen introduced[16]. Thus, there appears to be several outcomes possible, such that at low antigen doses there is no detectable response, and with increasing doses of antigen there may be the induction of tolerance, while even higher doses are necessary to trigger an immune response, which involves more and more antigen-reactive cells as the antigen dose increases. At very high antigen doses there may even be a "paralysis" induced.

Another key to understanding the immune response resides in the realization that the absolute frequency of potential antigen-reactive lymphocytes is very low before the introduction of antigen, on the order of 1 in a million cells up to 1 in 10,000 cells (i.e. 10^-6 ^to 10^-4^). However, after antigen selection, proliferative clonal expansion increases the frequency of antigen-reactive cells to as high as 1 in 10 cells, an astonishing 1–100,000-fold increase [17-20]. Thus, the decision by individual cells to proliferate in response to recognition of an antigen is the critical decision at the cellular level that controls the ability of the whole immune system to discriminate between self vs. non-self.

Until the discovery of mitogenic cytokines, it was assumed that antigens are solely responsible for stimulating proliferation. It is now known that there are antigen-activated cytokines with T cell Growth Factor (TCGF) activity that provide molecular signals that markedly stimulate cell cycle progression. Since the principle cytokine with TCGF activity driving T cell proliferation is the interleukin-2 (IL2) molecule[21,22], the molecular mechanism whereby IL2 promotes cell cycle progression is of utmost importance.

Accordingly, to understand how the immune system discriminates between self vs. non-self antigens, the IL2 molecule is first examined, particularly how IL2 promotes the proliferation of antigen-selected cells. Then, the cellular and molecular determinants of IL2 production and IL2R expression are traced. Ultimately, the maturation of T cells in the thymus must be considered, to understand how cells determine whether to produce IL2 or not, and whether to respond to it or not. Most of the discussion that follows is focused on T cells, but the general principles developed apply to B cells as well.

## The Quantal Nature of IL2-Promoted T Cell Proliferation

At the level of the individual cell, the proliferative response to IL2 is *quantal*, i.e. it is "all-or-none"[23,24]. However, to proceed beyond simply a description of this phenomenon, one must understand the molecular basis for the response of individual cells to IL2, so as to predict the behavior of the immune system as a whole.

**Figure 1:**
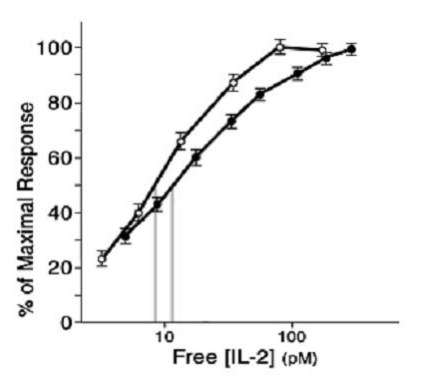
The IL2 binding and biological response curves are coincident. Radiolabeled IL2 and purified homogeneous IL2 were used in parallel experiments with the same IL2R+ T cell population to determine the relationship between IL2 binding and IL2-promoted T cell proliferation as monitored by ^3^H-TdR incorporation. From reference 25.

Soon after the development of the radiolabeled-IL2 binding assay[25], which permitted IL2 receptors to be quantified and defined for the first time, experiments could be performed to ascertain how the concentrations of IL2 that bind to IL2Rs compare with the IL2 concentrations that promote T cell proliferation. It was revealed that the binding and biological response curves are coincident, as shown in Figure 1[25]. The IL2 concentration-dependent response ranges from 1–100 pM, and the 50% effective concentration (EC_50_) equals the equilibrium dissociation constant (Kd) of the IL2-IL2R interaction, both of which are ~ 10 pM. Although this is a very high affinity for a ligand-receptor interaction (e.g. most TCR-peptide-MHC affinities are ~ a million-fold lower), it is noteworthy that in molecular terms, the EC_50_/Kd = 6 billion molecules/mL.

**Figure 2:**
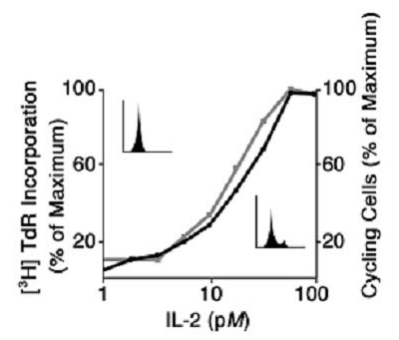
The IL2 biological dose-response relationship is determined at the single cell level by a marked heterogeneity of responsiveness. Asynchronously proliferating IL2R+ cells were exposed to varying concentrations of IL2 for 18 hours. Subsequently, cell aliquots were either pulsed for 4 hours with ^3^H-TdR, or stained with propidium iodide prior to single cell analysis by flow cytometry. The cell population incorporates ^3^H-TdR in an IL2 concentration dependent manner, and the amount of ^3^H-TdR incorporation at each IL2 concentration is determined by the absolute number of cells that have entered S-phase, as indicated by the single cell analysis by propidium iodide staining. From reference 26.

Of course, these experiments were performed using cell *populations*, and symmetrically sigmoid log-dose response curves are quite familiar in these circumstances. However, from these experiments it was not possible to discern whether the low thymidine incorporation found at low IL2 concentrations was due to *all *of the cells in population incorporating only a little thymidine, or whether only a few of the cells could respond at low IL2 concentrations. Therefore, it was not until the IL2 dose-response relationship could be examined at the *single cell *level did it become apparent that the cell populations are comprised of individual cells that differ markedly as to their responsiveness to the mitogenic ligand. Thus, as shown in Figure 2 using propidium iodide staining of DNA and the flow cytometer to compare with thymidine incorporation, it was found that some cells of an asynchronously proliferating population respond by proliferating to very low IL2 concentrations, e.g. only 1 pM, while others need 100-fold higher IL2 concentrations. Moreover, the marked heterogeneity in IL2 responsiveness could not be explained on a genetic basis, since even cloned cell populations behaved in an identical fashion.

Once it is realized that the effective IL2 concentrations span 2 orders of magnitude, the question becomes why some cells are capable of responding at only 1 pM, while others require IL2 concentrations that are 100-fold higher. The only logical answer to this question is that there must be intrinsic cellular differences, and that these differences are manifest in molecules that are critical for signaling cell cycle progression. Therefore, in experiments focused on understanding how IL2 promotes T cell proliferation after antigen activation, we found that in addition to the affinity of the IL2/IL2R interaction, and the concentration of IL2, the other variables involved are the IL2 receptor (IL2R) density, and the duration that the IL2 and the IL2R molecules interact[26].

In order to design experiments to approach these variables, it was necessary to use cell populations that were *synchronized *in early G_1_, so as to follow the *rate *at which individual cells progressed through G_1 _into S-phase. Thus, if G_0/1 _synchronized IL2R+ cells are exposed to two different IL2 concentrations, one receptor saturating and another only half-saturating; the receptor-saturating concentration promotes cell cycle progression twice as rapidly as the half-saturating IL2 concentration. However, as the ligand concentration dependency of cell cycle progression of cell populations was well known, these results were not surprising.

**Figure 3:**
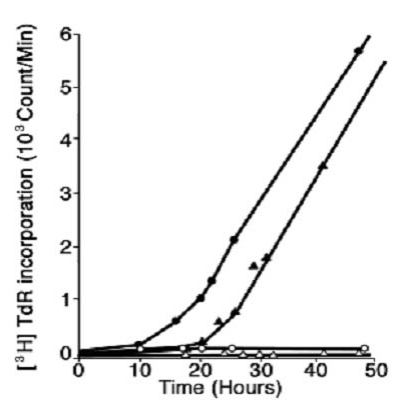
The effect of IL2R density on the rate of T cell cycle progression. Two G_0/1 _synchronized T cell populations that differed 3-fold in mean IL2R density were exposed to an IL2R saturating concentration (250 pM) for 48 hours and ^3^H-TdR incorporation was monitored as indicated at 1 hour intervals. The cells with the higher IL2R density (solid circles) entered S-phase before the cell population with the lower IL2R density (solid triangles). From reference 26.

However, it *was *surprising to find that if one exposes two cell populations, one with a higher IL2R density than another, to the same receptor saturating IL2 concentration, the cell population with the higher IL2R density traverses G_1 _and enters S-phase more rapidly than the population with the lower IL2R density (Figure 3).

**Figure 4:**
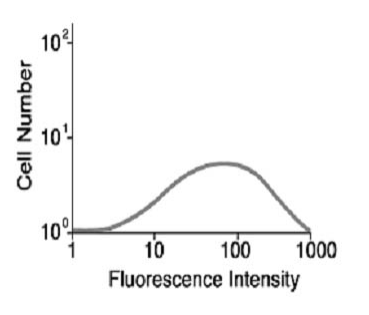
IL2R density determined at the single cell level by flow cytometry. IL2R+ T cells were labeled with anti-Tac (CD25) monoclonal antibody and analyzed by single cell flow cytometry. The IL2R density varies among cells within he population over 3 orders of magnitude. From reference 26.

These observations predict that the basis for the characteristic sigmoid IL2 log-dose response curve (Figure 1) is only explicable because of heterogeneity of IL2R density within a given population of T cells. The heterogeneity of IL2R density/cell is readily appreciated from examination of the flow cytometry plot of the log-normal distribution of IL2Rs as shown in Figure 4. Thus, at low IL2 concentrations, i.e. ~ 1 pM, only cells with the highest IL2R density are capable of responding. As the cell population is exposed to increasingly higher IL2 concentrations, cells with lower IL2R densities will meet the quantal requirement to enter the cell cycle. Finally, as the IL2 concentration reaches levels that saturate all IL2Rs, even cells with the lowest IL2R density can reach the critical number.

**Figure 5:**
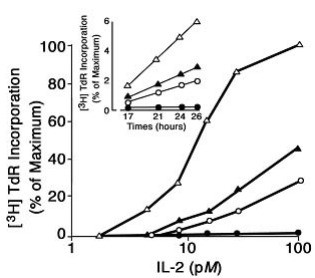
The effect of varying the IL2 exposure period on the proliferative response of G_0/1 _synchronized IL2R+ T cells. Aliquots of synchronized cells were exposed to IL2 for varying intervals (3, 6, 11, 26 hours) then washed and placed into culture without IL2 and pulsed with ^3^H-TdR. Symbols: IL2 exposure, 3 hr (solid circles), 6 hr (open circles), 11 hr (solid triangles), and 26 hr (open triangles). Inset, shows the ^3^H-TdR incorporation of each cell population in response to an IL2R saturating IL2 concentration (250 pM) monitored for 1 hr at the times indicated. From reference 26.

These findings indicate that cells reach a decision to undergo cell cycle progression based on some critical number of IL2-IL2R intermolecular reactions at the cell surface. Also, they indicate that the duration of the IL2-IL2R interaction plays a role, such that it appears that if a cell has a low density of IL2Rs, the critical number of IL2-IL2R interactions can still be reached, but a longer time interval is necessary. Also, the data indicate that if one interrupts the IL2-IL2R interaction before the critical number of interactions is attained, then cell cycle progression will not occur, as shown for a 3-hour exposure in Figure 5. In other words, the cell "*counts*" the number of triggered receptors and waits until the requisite number has accumulated before proceeding beyond G_1 _to S-phase[24,27].

IL2/IL2R binding is rapid and comes to steady state within 10 minutes[25,28], yet several hours of IL2 exposure are necessary to reach the critical number of IL2/IL2R interactions required to trigger cell cycle progression. Therefore, it must be concluded that the requisite number of IL2Rs is not present on any cells before the addition of IL2. Instead, new receptors must be continuously synthesized, expressed on the cell surface and *serially *engaged over several hours to finally reach the quantal number. Accordingly, the decision to divide is regulated with exquisite high fidelity, and the decision must be quantal. Otherwise a cell might only partially replicate its DNA before undergoing cytokinesis, a situation clearly incompatible with life.

An estimate of this critical number of IL2/IL2R interactions required can be calculated[24], knowing the initial mean number of receptors (~ 750 Rs/cell), and the rate of internalization and degradation of IL2 bound IL2Rs from the cell surface at steady state (t_1/2 _= 15 minutes). Thus, the rate constant, κ = ln2/15 min = 4.67 × 10^-2 ^min^-1^, governs the rate of new receptor synthesis necessary to maintain the surface expression at steady state. Moreover, 11 hours (660 minutes) of IL2 exposure is necessary to trigger 50% of the cells within the population to undergo cell cycle progression (Figure 5). Thus, the mean number of triggered IL2Rs necessary is:

R# (R/cell) = κ × R# @ steady state

= 4.67 × 10^-2 ^min^-1 ^× 750 R/cell

= 35 R/cell/min × 660 min

= 23,100 triggered R/cell

It follows that if the mean number of IL2Rs at steady state is lower, e.g. only 375 Rs/cell, 22 hours would be required to reach the quantal number and if the rate of new receptor synthesis is doubled, maintaining twice as many, 1,500 sites/cell, then only 5.5 hours would be required to trigger the cells.

## Intracellular Sensors of the Extracellular Signals

There are 3 chains that make up the trimeric IL2R that has an extremely high affinity for IL2, termed α (CD25)[29], β (CD122)[30] and γ (CD132)[31]. Careful kinetic and equilibrium binding experiments using radiolabeled IL2 revealed that the α chain contributes a very rapid association rate (κ = 10^7 ^M^-1^sec^-1^), while the β chain contributes a slow dissociation rate (κ' = 10^-4 ^sec^-1^), which together yield the high affinity (Kd = κ'/κ = 10^-11 ^M) for the ligand observed at steady state[28].

Recent experiments focused on the energetics of assembly of IL2/IL2R signaling complexes have revealed that in solution, the IL2R α and β chains bind to one another, whereas the α chain does not bind to the γ chain[32]. Moreover, the γ chain can only bind to α,β dimers or isolated β chains if IL2 has already bound to these receptor chains. These data support the interpretation that the α,β dimer probably is formed on the surface of antigen-activated T cells, and serves as the initial receptor complex capable of binding IL2. Moreover, the 10–20-fold excess expression of the α chain vs. the β chain favors the formation of the α,β heterodimer by the law of mass action. Subsequently, the IL2, α,β trimeric complex then can attract and bind the γ chain, forming a quaternary complex that is capable of signaling the cell interior. These energetic experiments provide an explanation for the IL2-dependency of signaling, since signaling only occurs when the γ-chain and the β chain are brought into close proximity, thereby activating the tyrosine-specific kinases, JAK 1 and 3, which are already bound to the β and γ chains respectively[33]. Accordingly, the assembly and maintenance of this energetically stable multicomponent macromolecular signaling complex is a fundamental requirement for the cell ultimately to make the quantal decision to divide.

The important downstream events in IL2-promoted T cell cycle progression are the JAK-dependent activation of at least two distinct proliferative signaling pathways. One is mediated by the transcription factor STAT5, while the other is mediated by the adapter molecule Shc, which activates phosphatidylinositol 3-kinase (PI3K). Both cyclin D2 and D3 are expressed in response to IL2R triggering[34], and recent studies have shown that the STAT5 and PI3K pathways play distinct, but coordinated roles in the quantal IL2R induction of progression through the Restriction Point (R-point) in the G_1 _phase of the cell cycle [35-37]. In addition to Shc, STAT5 also facilitates the activation of the PI3K pathway by a delayed mechanism that requires protein synthesis, and PI3K activity is essential for the induction of cyclin D2 expression by STAT5. PI3K activity is required for the optimal binding of RNA polymerase II to the promoters of cyclin D2 as well as other IL2/STAT5-induced genes.

Because of these findings, it has been proposed that the D cyclins serve as intracellular sensors of the extracellular signals [38] generated at the cell surface by the formation of the stable quaternary IL2/IL2R signaling complex[39]. Thus, cyclin D2 and D3 complex with the cyclin-dependent kinases (cdk) 4 & 6 and the p27 protein, thereby forming active kinases that initiate phosphorylation of the Rb proteins that repress the E2F transcription factors. The E2Fs are already bound to response elements that regulate the expression of multiple genes, the expression of each of which is critical for both nucleotide synthesis and DNA replication. Also, the E2F transcription factors regulate the expression of multiple genes required for formation of the Pre-replication Complexes (Pre-RCs), which must first assemble and then disassemble at sites on DNA termed Origins of Replication (Ori) before the DNA strands can separate, thereby allowing DNA duplication. Accordingly, the cyclin D/cdk/p27-dependent phosphorylation of Rb has been proposed to initiate the passage through the R-point, and has been described as a quantal molecular "binary switch"[39,40].

However, recent experiments with mice that have had all 3 of the cyclin D genes deleted have revealed that although hematopoiesis is dependent on the coordinated expression of the cyclin D genes, nonhematopoietic cells can proliferate in the absence of the D-type cyclins and their cyclin-dependent kinases 4 and 6 (cdk4/6)[41,42]. Even so, mouse embryo fibroblasts lacking type D cyclins proliferate more slowly to stimulation by serum by comparison to their wild type counterparts. Therefore, it appears that there are at least 2 distinct pathways whereby extracellular signals can trigger G_1 _progression, one involving cyclin D and another that is cyclin D independent.

At this time, it remains unknown as to whether T cells can be stimulated to proliferate by both cyclin D-dependent and independent pathways, or only by cyclin D-dependent (and therefore IL2R/STAT5-dependant) pathways. However, it is clear that T cells are in G_0 _until activated by initial signals received via the TCR, so that it still remains possible that T cells may be capable of proliferating in response to *both *TCR- and IL2R-derived signals[33,43,44]. Alternatively, the TCR may be responsible for the G_0 _to G_1 _transition, while the IL2R is responsible for G_1 _progression to the R-point and S-phase transition. However, another possibility remains that it still may well be that TCR-derived signals can initiate early rounds of cell division, but that for a fully developed clonal expansion, IL2/STAT5-dependent signals are necessary. If so, one would predict that TCR-mediated cell cycle progression is STAT5- and cyclin D-independent, so that the role of IL2 is to markedly accelerate and extend cell cycle progression initiated by the TCR.

## The Quantal Regulation of IL2 Gene Expression

Once one realizes that the quantal decision of T cells to proliferate is based upon a critical number of IL2-IL2R interactions, it becomes immediately obvious that the availability of IL2, together with the extent of IL2R expression, ultimately determines whether a immune response occurs that will be detectable at the systemic level. Since the discovery [45-51] and elucidation of the nature of the T Cell antigen Receptor (TCR) over the past 20 years [52-56], data have accumulated indicating that the regulation of IL2 gene expression and IL2R gene expression is under a tight and complex cell surface signaling mechanism that involves not only the TCR, but other surface molecules as well.

Before the elucidation of the structure and function of the receptors involved in antigen recognition, it was difficult to envision how the IL2/IL2R system is regulated. However, the principles from the quantal IL2-IL2R signaling of cell cycle progression can now be extrapolated to this more complex signaling system, and herein resides the ultimate control of "self-non-self" recognition and response. Like the IL2R[24,27], the TCR also is capable of counting the number of antigen interactions so as to acquire the critical number of triggered receptors necessary for IL2 gene expression.

Since the cloning and sequencing of the IL2 cDNA[57] and gene[58], detailed studies have revealed the nature of the molecules controlling IL2 expression. There are 3 distinct response elements in the promoter region of the IL2 gene that bind members of distinct families of transcriptional activating factors [59-61]. These factors include Activating Protein-1 (AP-1), Nuclear Factor of Activated T cells (NF-AT), and the Nuclear Factor kappa B/Rel (NF-κB).

Individual IL2 transcription factors from these three families cannot bind stably to their target DNA response elements *in vivo *without coengagement of each of the distinct factors that bind at neighboring sites[62]. Also, if the members of any one of these factors is prevented from binding to the IL2 promoter region, there is a marked attenuation of IL2 gene transcription[63]. Moreover, even after the factors have bound, inactivation of any of the three transcription factors pharmacologically extinguishes the binding of all three factors, thereby aborting transcription[64]. Therefore it has been proposed that there is a nonhierarchical, cooperative enhancement of binding at the IL2 gene locus, and that this binding and transcriptional activation of IL2 gene expression is consequently, quantal.

The quantal binding of IL2 transcription factors to the IL2 enhancer and promotion of IL2 gene expression has been extended by studies on the allelic expression of the IL2 genes. Under optimal TCR stimulating conditions, where antigen is in excess, and Antigen Presenting Cells (APCs) are not limiting, there is biallelic expression of the IL2 genes[65]. By comparison, when TCR stimulatory conditions are suboptimal, then expression can be monoallelic, and as well, fewer cells will be activated to express IL2. However, once engaged, the rate of IL2 production per cell remains constant. In this respect, IL2 gene expression per cell is always quantal.

The activation of NF-AT, AP-1 and NF-κB/Rel family members is controlled by signaling pathways triggered by the TCR, as well as costimulatory molecules and coinhibitory molecules. Thus, TCR triggering promotes the rapid influx of calcium into the cell, which activates the phosphatase calcineurin, thereby dephosphorylating and activating NF-AT[66], while other TCR-triggered pathways activate the kinases p56^lck^, ZAP-70, PLCγ, and Protein Kinase C-θ (PKC-θ), which simultaneously promote the activation and translocation of AP-1 to the nucleus, and the activation of NF-κB/Rel family members that are already present in the cytoplasm bound to the Inhibitors of κB (IκB)[67]. In addition, stimulation of co-stimulatory receptors, particularly CD28, by the B7 ligands expressed by APCs activates PI3K, thereby further activating both AP-1 and NF-κB/Rel members [68-70].

## The Phenomenon of Anergy ("Abnormal Inactivity")

Soon after the derivation of the first T cell clones[71], experiments with cloned helper T cells revealed that high concentrations of specific antigenic peptide (i.e. 1–100 μM) would lead to unresponsiveness, i.e the incapacity to produce IL2 or to proliferate when subsequently exposed to a stimulatory concentration of antigenic peptide (i.e.1 nM-1 μM)[72]. The suppressive effect was peptide and clone specific, took several hours to develop, and was long lasting, up to 7 days *in vitro*. It could not be ascribed to nonspecific toxicity and cell death, in that the cells were still capable of proliferating in response to IL2 added exogenously.

Subsequently, anergy (defined as a state of proliferative unresponsiveness to normal mitogenic activation) was shown to be produced by delivery of "Signal-1" (i.e. TCR), without "Signal-2" (i.e. CD28)[11]. More recently, it has been shown that it is possible to induce anergy pharmacologically by stimulating calcium flux using calcium ionophores without activating the TCR or CD28[66,73,74]. In this instance, NF-AT is activated and translocates to the nucleus in the absence of activation of AP-1 and NF-κB/Rel. Activation of NF-AT without the participation of AP-1 and NF-κB/Rel results in the proteolytic degradation of PLCγ and PKC-θ [74], as well as the transcriptional activation of a unique set of genes that collectively suppress the capacity of the cell to respond to subsequent full TCR/costimulatory activation[73]. This induced "anergic state" is stable and is manifest by the inability to maintain a stable immunologic synapse, which precludes expression of the IL2 gene, so that anergic cells will not proliferate in response to a subsequent full TCR/CD28 stimulation. However, like the high antigen dose anergy, if IL2 is supplied exogenously, the proliferative block is bypassed, so that the anergized cells are still capable of proliferating in response to the IL2 signals.

These findings are reinforced by other experiments, which reveal that inhibitors of calcineurin, such as cyclosporine-A and tacrolimus (FK506), which prevent the activation of NF-AT, also prevent the induction of anergy[75]. As well, T cells from NFAT-1 (-/-) mice are resistant to anergy induction by calcium ionophores[76,77]. Moreover, NF-AT-1 (-/-) mice exhibit a syndrome characterized by the accumulation of hyperactivated T cells[76,77]. Thus, in situations where calcium-mediated activation of calcineurin and NF-AT predominates, and pairing with AP-1 and NF-κB/Rel transcription factors does not occur, such as in situations with little of no costimulatory activation, a biochemical milieu exists that favors the creation of an anergic state.

By comparison, NF-κB/Rel appears to be the most critical of the three families of transcription factors involved in the activation of IL2 gene expression[78]. Moreover, of the 5 members of the NF-κB/Rel family, c-Rel is the most important, and also the critical transcription factor activated by costimulatory signals[79]. C-Rel expression is restricted to cells of the lymphoid and myeloid lineages, whereas the other NF-κB family members are expressed ubiquitously in almost all tissues. T cells from c-Rel (-/-) mice cannot express the IL2 gene or proliferate in response to activation via full TCR/co-stimulation. However, they can proliferate normally if IL2 is supplied exogenously[79]. As well, c-Rel binds to the costimulatory response element IL2 CD28RE with a high affinity (Kd = 25 nM), while NF-κB/Rel p50/p65 heterodimers bind to this response element with a 10-fold lower affinity[80].

Accordingly, for productive CD4+ T cell activation manifest by IL2 gene expression and proliferative clonal expansion, the minimum requirements are optimal activation of the TCR via peptide-MHC complexes, and costimulation via activation of CD28 by the APC B7 ligands. In the case of a low affinity TCR-MHC-peptide interaction, or in the absence of costimulation, NF-AT activation may predominate and anergy can result[81]. The actual critical number of TCR/CD28 activating signals that result in the quantal expression of the IL2 gene have not been determined, but by extrapolation from the parameters regulating IL2-IL2R activation, it is logical that peptide-MHC ligand concentration, TCR receptor density and affinity, as well as duration of the ligand-receptor interaction, will dictate the number of triggered TCR/CD28 interactions, which determine whether the cell becomes anergized vs. activated to produce IL2, to express IL2Rs and to proliferate.

## The Regulation of IL2R Gene Expression

Resting T cells that have not been recently activated via TCR/CD28 do not express detectable high affinity IL2Rs[25]. A recent study carefully examined resting T cells isolated from human peripheral blood for expression of the 3 chains of the IL2R by flow cytometry[82]. To ensure that the T cells represented only unactivated, truly "resting" T cells, any *in vivo *activated cells were removed using monoclonal antibodies reactive with the transferrin receptor (CD71), known to be an early TCR/CD28 activation molecule. Both CD4+ and CD8+ resting T cells have undetectable surface or cytoplasmic IL2R α and β chains, as monitored using very sensitive flow cytometry methods. By comparison, IL2R γ chains are detectable in the cytoplasm, but undetectable on the cell surface[83]. Upon activation via the TCR/CD28, the expression of the genes encoding both the IL2R α and β chains occurs, and γ chains are rapidly mobilized to the cell surface, so that high affinity trimeric IL2Rs are expressed, and the cells are competent to respond to IL2 by proliferating.

The regulation of α chain (CD25) gene expression is under the control of the TCR/CD28 via activation of NF-κB/Rel, AP-1 and NFAT, which interact with 2 distinct REs [84]. Therefore, it appears that the IL2Rα chain gene is coordinately regulated along with the IL2 gene by the same signaling pathways emanating from the TCR/CD28 receptors that activate the same families of transcription factors regulating the IL2 gene. However, it has not been determined whether the critical number of TCR/CD28 receptors necessary to trigger the IL2 gene and the IL2Rα chain gene are similar. Most experience suggests that there is a much lower number of triggered TCR/CD28 receptors regulating IL2Rα gene expression as compared with IL2 gene expression, but this question needs to be examined directly.

In addition to the TCR, IL2 enhances IL2Rα chain gene expression as much as 10–20-fold [85-87]. This IL2 effect on α-chain expression is readily appreciated by flow cytometry, in that IL2 shifts the mean fluorescence intensity more than an order of magnitude. As well, it is noteworthy that the IL2Rα chain expression is ~ 10-fold higher than expression of either the IL2Rβ or the IL2Rγ chains, so that when using flow cytometry to detect each of the chains, the IL2Rα chain (CD25) is always predominant. As well, the number of functional high affinity trimeric IL2Rs is determined by the number of β and γ_c _chains, which are limiting. The energetics of IL2 chain assembly has now provided an explanation for the excess α chains, in that the law of mass action favors the formation of α,β heterodimers, to which IL2 binds and then recruits the γ chain, thereby forming the stable quaternary signaling complex.

## The *In Vivo *Functions of IL2

Before the identification of the IL2 molecule in 1983[57,88], it was assumed that antigens stimulate T cell proliferation, and that mitogenic cytokines, which were first described almost 20 years earlier[89,90], functioned simply to amplify the signals already initiated by antigen[22]. Thus, when purified IL2 became available[88] and the IL2 receptor (IL2R) had been discovered[25], experiments became possible for the first time to determine the molecular mechanism whereby antigen activated T cells are stimulated to proliferate. Initial studies revealed that although antigen is necessary to activate T cells to leave G_0 _and to enter early G_1_, cell cycle progression through G_1 _to S-phase and mitosis appeared to be mediated by IL2 upon binding to the IL2R[26,91,92]. Resting T cells were found to be IL2R negative and IL2 unresponsive[25], while purified, homogeneous IL2 was capable of promoting long-term T cell proliferation of mitogen- or antigen-activated T cells. By comparison, mitogen or antigen alone could not sustain long-term T cell growth[26].

Moreover, immunosuppressive pharmacological agents such as glucocorticoids[93] were found to inhibit T cell proliferation by preventing IL2 production but not IL2 responsiveness. As well, experiments with monoclonal antibodies that block either IL2[88] or the IL2R[29] were found to inhibit T cell cycle progression after mitogen or antigen activation. These experiments all suggested that antigen *per se *could not promote T cell proliferation, and suggested that IL2 drives T cell proliferation after the initial antigen activation. Even so, the monoclonal IL2- or IL2R-reactive antibodies suppressed proliferation by > 90–95%, but never completely abrogated proliferation, leaving open the possibility that either the TCR itself or other mitogenic cytokines might also be operative.

In 1991, with the advent of IL2 gene deletion through genetic recombination, it became possible to test definitively the functional importance of IL2, both *in vitro *and *in vivo*, at least in mice. Initial *in vitro *experiments testing cells from IL2 (-/-) mice for proliferation in response to activation by the mitogenic lectin Concanavalin-A (Con-A) revealed a 70–75% diminution of tritiated thymidine incorporation, but not a complete abrogation[94]. Therefore, these experiments suggested that perhaps the TCR really was capable of promoting proliferation, and that IL2 functioned to merely amplify the response, as assumed originally. Alternatively, it was also considered possible that perhaps IL2 was not the only cytokine with TCGF activity operative to promote T cell cycle progression, and additional cytokines such as IL4[95] or IL7[96], which were thought originally to be primarily B cell stimulators, might also be playing roles.

Even so, it was still a great surprise when initial *in vivo *experiments with IL2 (-/-) mice infected with Vaccinia Virus (VV) and Lymphocytic Choriomeningitis Virus (LCMV), revealed that the generation of antigen-specific effector cytolytic T cell activity was reduced by only ~ 30% as monitored by Cytolytic T Lymphocyte (CTL) ^51^Cr-release assays[97]. Moreover, neutralizing IgG antibody responses to Vesicular Stomatitis Virus (VSV) infection, a T-helper-dependent function, were delayed but not reduced. Other *in vivo *experiments with staphylococcal super antigens indicated that CD4+ T cells doubled normally, while CD8+ T cells from IL2 (-/-) mice were only ~ 50% of wild type[98]. These findings led to the interpretation that *in vivo *IL2 is redundant for the generation of immune responses, and that the TCR or other cytokines with TCGF activity could substitute for IL2.

However, upon subsequent and more extensive testing of IL2 (-/-) mice, it was found that in the absence of IL2, the marked proliferative expansion of LCMV-induced CD8+ T cells was virtually eliminated, the total cytolytic effector capacity was reduced by > 90%, and IFN-γ production resulting from T cell activation was dramatically inhibited[99,100]. Moreover, IL2 (-/-) mice permitted prolonged viral replication compared with (+/+) and (+/-) controls, which could clear the virus within a few days.

Therefore, all of these data indicated that IL2 may not be the sole cytokine with TCGF activity, but it is one of the principle TCGFs responsible for the maximal proliferation of antigen selected mature peripheral T cells, as well as their differentiation to effector cells, both *in vitro *and *in vivo*. Moreover, IL2 (-/-) mice are immunocompromised without IL2; thereby indicating that the TCR or other yet undiscovered cytokines cannot fully substitute for IL2 *in vivo*.

## The IL2 Deficiency Autoimmune Syndrome

Since IL2 (-/-) mice are immunocompromised, it was entirely unexpected to find that a syndrome of lymphocyte hyperactivity and apparent autoimmunity appears as the mice mature beyond puberty [101]. Thus, although lymphocyte development during embryogenesis is grossly unperturbed by the absence of IL2, generalized lymphoid hyperplasia ensues after the first several weeks and months of life, and T cells that express activation markers accumulate in the secondary lymphoid tissues. As well, autoimmune antibody-mediated hemolytic anemia appears, in addition to antibodies reactive to self-molecules, such as DNA and other nuclear antigens. Similar findings with mice deleted of the IL2R α chain (CD25)[102], β chain (CD122)[103], the JAK3 protein kinase[104,105], and the transcription factors STAT5a/b[106] all supported the idea that IL2 or one of the other interleukins that signal via the IL2R γ_c _chain [107] somehow determines the selection of mature cells in the thymus, and in the absence of postnatal IL2 expression, the immune system begins to react to self as if it is nonself.

The accumulation of T cells with an activated phenotype in the setting of an initial lymphopenia and immunodeficiency, has recently been attributed to compensatory over stimulation by the cytokines responsible for homeostatic proliferation, e.g. IL7, IL15 and IL21, all of which signal via the γc chain[108]. Of these cytokines, IL7 and IL15 signal via STAT5, whereas IL21 signals via STAT1 and STAT3. Accordingly, IL21 stimulation via STAT1&3 may well be responsible for the hypersensitivity lymphoproliferative syndrome that is common to the IL2, IL2R and signaling (-/-) phenotype.

These observations in mice were reinforced by a report of a human homozygous mutation of CD25[109]. A male child of first cousin parentage presented at age 6-months with increased susceptibility to viral, bacterial, and fungal infections, suffering from cytomegalovirus pneumonitis, persistent oral thrush, candida esophagitis, and adenovirus gastroenteritis, chronic diarrhea, and failure to thrive. From the age of 8-months, lymphadenopathy and hepatosplenomegally became apparent. *In vitro *assays demonstrated a reduced responsiveness to stimulation by anti-CD3 (11% of control) and phytohemagglutinin (20% of control). Severe immunodeficiency was proven by the patient's inability to reject an allogeneic skin graft. Paradoxically, despite the obvious immunodeficiency, there was a normal sized thymus and lymphocytic infiltration of multiple tissues, including lung, liver, gut, soft tissue and bone. These findings were interpreted as possibly a result of a failure of negative selection of potential autoreactive cells in the thymus, as well as an inability to control autoreactive cells in the periphery, perhaps due to the absence of CD4+CD25+ Regulatory T cells.

## Regulatory T Cells (T-Regs)

Soon after it was demonstrated that IL2 (-/-) and IL2Rα or β chain (-/-) mice develop an autoimmune syndrome, it was reported that immunocompromised (*nu/nu*) mice would also develop a wide spectrum of both organ-specific and systemic autoimmune diseases if they received normal cell populations from which CD4+CD25+ T cells were eliminated[110]. Furthermore, reconstitution of CD4+CD25+ T cells in the transferred cell populations prevented the development of autoimmunity. Subsequently, we found that IL2 treatment of IL2 (-/-) mice before day 10 after birth prevents the onset of the syndrome of lymphocyte activation and autoimmunity[111]. As well, thymocytes and spleen cells from IL2-treated IL2 (-/-) mice transferred to IL2 (-/-) recipients delayed the development of the autoimmune syndrome. These data suggested that IL2 treatment induced some normal cellular maturation/differentiation step in the transferred IL2 (-/-) cells that subsequently prevented the cells in the IL2 (-/-) mice from responding to self-antigens[111].

In other reports from as early as the 1960s[112], it was established that neonatal thymectomy in the first few days after birth can lead to subsequent widespread autoimmune phenomena, such as hemolytic anemia, thyroiditis, gastritis, oophoritis, or orchitis [113-115]. These two observations, i.e. autoimmune phenomena arising in both IL2 (-/-) mice and neonatal thymectomized mice, were connected when it was demonstrated that T cells expressing the IL2Rα chain (CD25) ontogenically begin to appear in the normal periphery immediately after day 3 of life, rapidly increasing within 2 weeks to adult levels, which comprise ~ 10% of CD3+ cells [116]. As well, neonatal thymectomy on day 3 eliminates CD25+ T cells from the periphery, and injection of CD25+ T cells from normal adult donors into day-3 neonatally thymectomized mice prevents the development of autoimmunity, while injection of CD25- T cells does not.

These observations were interpreted as consistent with the notion that neonatal thymectomy on day 3 can eliminate or reduce the autoimmune preventative CD25+ T cells, thereby leading to unchecked activation of the self-reactive T cells produced before neonatal thymectomy. Together with the observations on the IL2 treatment of IL2 (-/-) mice[111], these experiments fix the source of these CD25+ cells within the thymus, and also imply that IL2 is a necessary component in their development.

CD4+CD25+ "suppressor" regulatory T cells (T-Regs) could also be demonstrated in the secondary lymphoid organs of normal adult mice, and an *in vitro *assay was devised to test their suppressive activity[117]. CD4+CD25+ cells typically represent ~ 10%–15% of CD4+ T cells in lymph nodes from 8–10 week old mice. Paradoxically in view of their expression of CD25, these cells appear to be resting as well as anergic, in that they cannot proliferate in response to soluble and solid-phase anti-CD3 or Con-A. As well, even though the cells express the IL2R α chain, they cannot proliferate in response to exogenous IL2. However, these cells can be activated and proliferate in response to a *combination *of anti-CD3 + IL2. These data suggest that resting, anergic CD4+CD25+ T cells do not express the IL2R β and γ chains, but they can be induced to express them after activation of the TCR.

When co-cultured with CD4+CD25- cells, these CD4+CD25+ cells were found to markedly suppress the proliferative response of the CD25- cells, provided they are stimulated with low concentrations of soluble, but not solid-phase anti-CD3. This inhibition was dependent on cell-cell contact, not soluble factors, and dependent on the suppression of IL2 production by the CD25- responding cells. The inhibition could be bypassed by the addition of IL2, or costimulation with anti-CD28. These data were interpreted as consistent with the idea that CD4+CD25+ cells in normal unimmunized animals represent a distinct lineage of "professional" suppressor cells that have matured in the thymus. These T-Reg cells have been termed "naturally occurring" (nT-Reg).

It has also been reported that CD4+ T cells with regulatory function can be generated *in vitro *by the activation of mature peripheral CD4+CD25- T cells[118]. Thus, both regulatory and effector cells can, in principle, be generated from the same mature CD4+ T cell precursors. It has been postulated that these "inducible" cells (iT-Regs) might be triggered by "low-affinity or altered TCR signal transduction"[118], in that the conditions that favor the generation of T-Regs *ex vivo *from mature CD4+CD25- T cells, include antigen in the presence of immunosuppressive cytokines such as IL10 and TGFβ, immunosuppressive agents such as vitamin D3 and dexamethasone, CD40-CD40L blockade or immature DC populations[119,120].

Furthermore, it was reported recently that subcutaneous infusion of low doses of antigenic peptide by means of osmotic pumps over 14 days transforms mature peripheral T cells into CD4+CD25+ "suppressor cells" that can persist for long periods of time (i.e. several months) in the absence of antigen and confer specific immunological tolerance upon challenge with immunogenic doses of antigen[121]. Therefore, it appears that both *in vitro *and *in vivo*, low antigen concentrations can promote the differentiation of mature peripheral CD4+ T cells to express CD25, and to become "suppressor" cells rather than effector cells.

The dependency on the thymus for maturation of CD4+CD25+ T-Regs, as well as the dependency upon IL2, has been underscored by experiments with IL2β chain (-/-) mice made transgenic for the IL2Rβ chain under the influence of the proximal *lck *promoter, so that mature trimeric IL2Rs capable of signaling are only expressed in the thymus[122,123]. Transgenic expression of the IL2Rβ chain in IL2Rβ chain (-/-) thymocytes corrects the lack of CD4+CD25+ peripheral T cells in IL2Rβ chain (-/-) mice and prevents lethal autoimmunity. These experiments further emphasize the unique contribution of IL2 to the development of CD4+CD25+ T-Regs, in that the other γ_c _chain cytokines, e.g. IL4 and IL7, IL9, and IL21 do not compensate for the lack of T-Regs in IL2Rβ chain (-/-) mice.

Suboptimal antigen concentrations and IL2 are both required for the generation and activity of T-Regs, and both in the thymus and in the periphery. However, the forces governing the simple generation of anergic and nonfunctional CD4+CD25+ T cells, as compared with the determinants of the generation of activated proliferating, suppressive CD4+CD25+ "Regulatory T cells", vs. CD4+CD25+ activated proliferating functional effector T cells remain to be defined.

Because each of these cell fates is functionally distinct, it is reasonable to hypothesize that these distinct cell fates are determined by different numbers of triggered TCRs and IL2Rs.

## The Transient Nature of the *In Vitro *T Cell Proliferative Response: Feedback Inhibition of IL2 Gene Expression

Upon productive activation of T cells *in vitro *via TCR/CD28, there is a characteristic transient expression of the IL2 gene, such that IL2 mRNA first becomes detectable within 6 hours, and then peak levels occur after 12 hours, with a subsequent decline to undetectable levels by 24 hours[21]. Detectable IL2 protein in the culture media follows a similar, but delayed course with peak concentrations found after 24 hours, and by 48 hours barely detectable levels remain. Expression of high affinity trimeric IL2Rs follow a similar, but delayed transient expression course, with peak levels expressed at 24–48 hours, followed by a slow decline over several days[91]. By comparison, expression of the IFN-γ gene follows a much more protracted course, with detectable expression still evident after several days[124].

The mechanisms accounting for the transient expression of the IL2 gene and the genes encoding the IL2R chains have not been apparent, until recently. It is now realized that surface molecules of the coinhibitory CTLA-4 family[125] appear later after TCR/CD28 activation, first evident after ~ 24 hours with peak levels at 48–96 hours. In addition to CTLA-4, which has a 10-fold higher affinity for the B7 molecules expressed by APCs, the coinhibitory receptor PD-1[126], as well as the ligands reactive with this receptor, PDL-1[126] and PDL-2[127], appear on activated T cells. Still a third, later appearing coinhibitory receptor, BTLA, has also recently been described as expressed on both antigen-activated T cells and B cells[128].

This coinhibitory ligand-receptor family of molecules belongs to the Ig superfamily and the B7/CD28 costimulatory ligand/receptor family[129,130]. However, the CTLA-4 family of receptors does not function to deliver a costimulatory signal, as does CD28. Instead, members of the CTLA-4 family have inhibitory signaling motifs in their cytoplasmic domains and they have been shown to localize in close proximity to CD28, where they compete with activating signals from CD28. For example, by binding the phosphatase SHP-2, the CTLA-4 family of molecules inhibits the positive signals emanating from the TCR/CD28 at a very proximal position within the signaling cascade, thereby extinguishing the expression of the IL2 gene. Once IL2 production is shut down, because IL2 is internalized and degraded with a half-time (t_1/2_) of ~ 2 hours, IL2 is consumed very rapidly, resulting in cessation of IL2-promoted cell cycle progression and eventually apoptosis due to the lack of IL2-promoted anti-apoptosis gene expression, such as Bcl-X_L_.

The coinhibitory ligand/receptor pairs identified thus far can be shown to exert their negative effects by attenuating IL2 gene expression, but the administration of IL2 can bypass this block, in that the cells are still IL2-responsive. A similar phenomenon has been found with regard to the effects of T-Regs. T-Regs shut down IL2 gene expression, via a cell-cell contact mechanism that has not yet been delineated. However, IL2 supplementation will bypass the suppressive effects of T-Regs, thereby allowing for T cell proliferation. Also, in both instances, activation of CD28 via monoclonal antibodies serves to counteract the blockades, and permits the suppressed cells to both express the IL2 gene and to proliferate.

Accordingly, it would seem a plausible hypothesis that the cell-cell contact mechanisms employed by T-Regs to inhibit IL2 gene expression are mediated by members of the CTLA-4 family of coinhibitory ligands/receptors, either those already identified, or others yet to be identified. This is a particularly attractive hypothesis, in that the ligands that trigger the coinhibitory PD-1 receptor are also expressed by TCR/CD28-activated T cells[131].

## The Transient Nature of the *In Vivo *T Cell Proliferative Response And "Adaptive Tolerance"

With the advent of the ability to label cells with 5- and 6-carboxyfluorescein diacetate succinimidyl ester (CFSE), combined with the use of gene deleted mice, it has been possible to follow the proliferation of T cells *in vivo*, and to test which proliferative signals are operative. In this regard, T cells from IL2Rβ (-/-) mice that have had the IL2Rβ chain expressed only during thymopoiesis do not express detectable IL2Rβ chains in the periphery, and therefore are incapable of delivering a proliferative signal. However, these cells are capable of undergoing 1–2 divisions upon stimulation with anti-CD3 + anti-CD28[132]. Similar findings have been reported in systems using TCR transgenic mice and adoptive transfer experiments when either IL2 or CD25 have been deleted[133,134]. One interpretation is that TCR/CD28 activation may be capable of initiating 1–2 rounds of cell division, but IL2 appears necessary for maximal and sustained proliferation. Alternatively, other, yet undiscovered cytokines with TCGF activity may be responsible for activating the initial proliferative responses to antigen activation.

After the initial proliferative clonal expansion of antigen-selected cells, the fate of the expanded effector cells has been found to depend greatly upon whether the antigen is cleared or whether it persists. In experimental viral infections where the virus is cleared rapidly within the first week after infection, expanded CD4+ and CD8+ effector cell populations undergo a contraction, with the loss of as much as 90% of the expanded effector cells[18]. We have found that this contractive phase is attributable to cytokine withdrawal apoptosis, in that the administration of IL2 during this phase prevents the contraction[135]. Others have shown a similar protective effect of IL2 after both CD4+ and CD8+ cells are expanded in response to activation by staphylococcal superantigen[136]. Subsequent studies have revealed that the residual populations of expanded effector cells eventually differentiate to "central" memory cells, which have the capacity for maintenance of the population size via slow proliferative renewal and as well, the capacity to respond to the reintroduction of antigen by rapidly producing IL2, proliferating and differentiating to effector cells[137].

With persistence of antigen, the fate of the expanded effector T cell populations changes dramatically. Instead of differentiating into responsive memory cells, the cells revert to a state of unresponsiveness, which has been termed "exhaustion" by those studying experimental persistent viral infections [138,139]. This exhausted state is manifested by an early loss of the capacity to produce IL2 and to proliferate. As antigen persists, the cells gradually lose their capacity to lyse target cells and to secrete antiviral effector cytokines such as TNF-α and IFN-γ. Eventually, clones of virus-specific cells can undergo apoptosis, which may be attributed to Activation-Induced Cell Death (AICD), leading to clonal disappearance[140].

A similar phenomenon has been described in experiments employing a paired transgenic model (TCR and Ag)[141]. In this model, CD4+ TCR-Tg T cells from antigen-naïve animals are labeled *in vitro *with CFSE, then transferred into recipient mice expressing low levels (~ 100 pM) of antigenic pigeon cytochrome *c *peptide. The cells become activated, express the IL2R α-chain (CD25) and CD69, and proliferate for the first 4 days, eventually expanding ~ 100-fold. However, over the subsequent 10–14 days, the cells lose expression of CD25 and cell recovery declines by ~ 50%. Thereafter, the cells are said to be in the "adaptive phase", which is characterized by a hyporesponsiveness to antigen *in vitro*, and is manifest by a decreased capacity to produce IL2 and other cytokines, and to proliferate.

This adaptive state persists in the host with chronic expression of the antigen, and in contrast to a similar paired transgenic model in a B cell system[142], is not associated with a decrease in the level of expression of the TCR[143]. However, the adaptive state is dependent upon continuous exposure to antigen; upon transfer to an antigen-negative host, the hyporesponsive state reverts. Moreover, the adaptive state is similar to a desensitization phenomenon akin to tachyphylaxis, and studies have revealed a down regulation of the TCR signaling molecules, involving an early block in tyrosine kinase activation, which primarily inhibits calcium mobilization, thereby suggesting that the desensitization involves the adaptation of the TCR signaling apparatus to the chronic persistence of low levels of antigen[143,144].

It is important to emphasize that the adaptive state is only a *relative *hyporesponsiveness, as compared to either the naïve situation or the host with central memory cells[141,143]. If cells are exposed to higher concentrations of peptide *in vitro*, i.e. between 1 nM and 1 μM, a response can be detected, but the antigen dose-response curve is shifted 100–300-fold to the right. In addition, the adaptive phenomenon cannot be ascribed to active suppression by a T-Reg differentiative process, in that the adapted cells do not express CD25, and in vivo experiments have excluded a suppressive mechanism.

## What Determines the "Strength" of the Signal?

From the discussion thus far, it must be apparent that the strength of the signals delivered to T cells ultimately determines the outcome, i.e. either anergy, or activation of the IL2 gene, and if activation occurs, the duration that it persists. Thus, considerations of the ligand concentrations available, the receptor affinities and numbers expressed, and the duration of the ligand-receptor interactions again become important.

The duration of signaling via TCR/CD28 is known to be a major determinant of the magnitude of IL2 production and thus the extent of T cell proliferation. Even in the 1970s the magnitude of the proliferative response after mitogenic lectin administration was found to be directly related to the duration of lectin stimulation[145]. Thus, removal of the mitogenic lectin within the first 24 hours of stimulation attenuates the proliferative response markedly.

With the discovery that T cell proliferation after mitogen or antigen activation is principally mediated by IL2[22], it seemed obvious that a certain time interval was necessary after TCR/CD28 triggering to provide for maximal expression of the IL2 and IL2R genes. Indeed, experiments proved this to be the case[146]. Even so, several hours seemed somewhat prolonged, given that early biochemical events such as calcium flux and kinase activation were detectable within minutes after TCR engagement. Moreover, IL2 gene transcription could be detected within 45 minutes, using sensitive techniques[147].

A series of experiments reported in the mid 1990s began to provide an explanation for this perplexing problem. By equating "triggered" TCRs with their internalization and disappearance from the cell surface after ligand activation, it was shown that a single peptide-MHC complex is capable of serially triggering up to ~ 200 TCRs[148,149]. Furthermore, like the IL2Rs, T cells appeared to be able to "count" the number of triggered TCRs, and responded by proliferating when ~ 8,000 TCRs were triggered[149,150]. Other experiments showed that the duration of antigenic stimulation was one of the most critical parameters determining the fate of naïve and effector T cells, i.e. whether they would be activated or deleted[151]. However, these observations were not linked to the IL2/IL2R system.

With the discovery of Supramolecular Activation Clusters (SMACs), an additional level of complexity was added[152]. Also termed the "Immunological Synapse", the specialized junction between a T cell and an APC consists of a central cluster of T cell receptors together with costimulatory and coinhibitory receptors, surrounded by a ring of adhesion molecules[153]. Recent experiments, which employed peptide antigen-specific TCR αβ transgenic T cell blasts labeled with CFSE, the relationship between the duration of the TCR signal and the extent of the proliferative response could be examined further at the single cell level. Between 10–24 hours of continuous TCR stimulation by MHC-peptide in a stable SMAC is necessary to promote maximal IL2 production and proliferation[154].

Regarding the density of the TCR on responding T cells, it is important to restate and emphasize that the TCR density follows the same log-normal distribution as detailed for the log-normal distribution of IL2Rs, even on cloned T cells. Thus, there is at least a 2-log_10 _difference in TCR density among potential responding T cells, and consequently, those cells with the highest density of TCRs, will be capable of responding to lower concentrations of MHC-peptide epitopes, and also capable of responding more rapidly than cells with lower TCR densities to an optimal pMHC concentration. Detailed studies examining the cytokine response of murine T cell clones to graded peptide antigen concentrations have revealed a hierarchical organization of TCR signal-dependent response thresholds for elicitation of different cytokines in individual cells[155]. IL2 production was found to remain constant per cell as the ligand concentration was increased, with the primary change being in the number of cells making IL2 at a fixed level. Therefore, the decision to produce IL2 is a quantal decision on the part of each individual cell within the cloned population.

Exactly what leads to the heterogeneity of the quantal response of IL2 gene expression on the part of the cells within the cloned cell population has not been determined, but very similar findings have been reported for IFN-γ production by a human T cell clone in response to graded doses of antigenic peptide[156]. The peptide dose that stimulated 5% vs. 95% of the T cells was found to span over a log, and the response on the part of the cells comprising the population was quantal; i.e. at low antigen concentrations fewer cells expressed IFN-γ and as the antigen concentration was increased, an increasing number of cells expressed IFN-γ. To explain these results it was postulated that the intraclonal heterogeneity in antigen responsiveness could result from the different numbers of TCRs expressed by individual cells. However, this conjecture has not been examined directly.

Exactly the same considerations hold for the distribution of costimulatory and coinhibitory molecules. Thus, there is interplay between all of these receptors, which ultimately impacts the "strength" of the signal[157] and the duration that the signal must be applied to productively signal the IL2 gene response elements, and eventually lead to an "activated" T cell. Obviously, if any of these parameters are limiting, the activation events may favor the delivery of abortive transcriptional activating signals, which may favor anergy or differentiation to T-Regs, rather than activation[141].

As already detailed, if CD4+ T cells are activated via the TCR without adequate stimulation via CD28, such as may occur if self peptide is presented on immature dendritic cells as APCs, the conditions would favor the predominant activation of NF-AT yet inadequate AP-1 and NF-κB/Rel activation, which would promote anergy and perhaps even the irreversible differentiation of most of the cells to T-Regs. Thus, the concentration of antigen, the availability of adequate costimulatory molecule function, the affinity and density of the TCR/cell, as well as the duration that the TCR is triggered all influence signal "strength".

With regard to the affinity of the TCRs for the peptide-MHC complex, it is important to note that the TCR does not undergo somatic hypermutation and "affinity maturation" as does the BCR. Accordingly, the Kd of the TCR is fixed after recombination and rearrangement in the thymus, and is relatively low by comparison with the Kd of antibody molecules, which have ~ 1000-fold higher affinity for binding antigen. Measurements of the equilibrium dissociation constants of isolated agonist pMHC binding to TCR molecules by surface plasmon resonance have revealed Kds in the range of 1–100 μM, with k_off _rate constants of ~ 0.01–0.1 s^-1^, yielding t_1/2 _~ 7–70 secs[158]. In contrast, antagonistic MHC-peptide-TCR interactions off-rates are ~ 10-fold faster than agonistic interactions. Thus, off-rate constants of ~ 5 sec^-1 ^have been found, which yield t_1/2 _of only ~ 0.15 seconds.

Of course, the TCR does not bind MHC-peptide complexes in isolation or in solution. The formation of the immunological synapse greatly alters the way in which TCRs engage antigens and the way in which they are triggered[55]. Thus, T cell clones that have TCRs with a Kd = 1 μM binding to MHC-peptide in isolation can be triggered at peptide concentrations ranging as much as 100-fold lower *in vivo*. Furthermore, new studies have made it possible to "count" the exact number of ligands that a T cell encounters on another cell, and then monitor the consequences of that interaction with respect to the increase of intracellular calcium concentration[55]. It has been found that only 10 MHC-peptide ligands are sufficient to provide for the formation of a stable immunological synapse and sustained calcium flux for several hours. However, below this critical number of MHC-peptide ligands, only transient calcium increases occur, and a stable synapse does not form. Thus, an abortive signaling process appears to ensue, which could very well lead to the activation of NF-AT without adequate levels of AP-1 or NF-κB/Rel, which would be insufficient for IL2 and IL2Rα chain gene expression, thereby promoting anergy/T-Reg differentiation.

Recent experiments focused on how the TCR can respond to such low concentrations of agonist peptides indicate that the slower off rate of the agonist pMHC/TCR interaction allows the juxtaposition of CD4 with bound *lck *to the agonist pMHC/TCR, so that endogenous pMHC/TCR, which are in a large excess, can form a dimeric signaling complex comprised of agonist pMHC and endogenous pMHC[159]. Then, the endogenous pMHC with its fast off rate can trigger many TCRs serially and greatly amplify the TCR signals.

Given the long duration necessary to trigger a response, a kinetic model has been proposed to account for how serially triggered TCRs that interact very briefly with peptide-MHC complexes, then are rapidly internalized and degraded can be counted by the T cell, and how transient signaling events can be accumulated over time and integrated into a quantal response[160]. The model is based on a process first described in neuronal cell activation termed 'temporal summation'. The signaling events originating from successively triggered TCRs build up, with each adding to the falling phase of the one before. In this way, small and short signals that alone are unable to trigger a response can be summed up over time eventually to reach the level sufficient to trigger the quantal response, in this instance IL2/IL2R gene expression.

All of these considerations lead one to the conclusion that like the IL2/IL2R-determined quantal decision to undergo cell cycle progression, there appear to be quantal decisions operative at the level of the immunological synapse that lead to distinct cell fates, which in turn are ultimately determined by IL2 and IL2R gene expression. Thus, if the agonist pMHC ligand concentration is low, only T cells with a high density of TCRs will form stable synapses that will result in sustained activation of the IL2 gene and thereby cell cycle progression. It follows that at the same limiting agonist pMHC concentrations, cells with lower TCR densities may have abortive expression of the IL2 gene, which would favor differentiation to T-Regs, while cells with still lower TCR densities would not successfully trigger expression of the IL2 gene, thereby favoring the triggering of differentiation to an anergic state and unresponsiveness. Thus, there are at least 3 distinct cell fates that are determined by the accumulated number of triggered TCRs, which is determined by the agonist pMHC concentration, TCR density and the duration of the pMHC/TCR interaction.

## The Quantal Numbers of Triggered Receptors are Specified in the Thymus

The structure and function of the immunological synapse essentially determines the fate of T cells as they mature in the thymus. Again, this cell fate determination is linked to IL2 and IL2R gene expression. From the above discussion, it is now clear that like the quaternary IL2/IL2R complex, the immunological synapse is a dynamic multicomponent molecular complex, the stability of which requires ongoing signaling through the TCR for stable calcium mobilization and kinase activation occurring over several hours. As well, the immunological synapse modulates the overall level of mature T cell activation by integrating positive (costimulatory) signals and negative (coinhibitory) signals from a variety of surface receptors. In this regard, it is noteworthy that the synapse that forms between thymocytes and thymic stromal cells differs qualitatively from that observed between mature peripheral T cells and peripheral APCs[161]. One reason that this may occur relates to the lack of expression of the costimulatory B7 molecules on thymic stromal cells[162]. In this regard, on would predict that thymocytes would not be activated to produce IL2 very readily, due to the lack of CD28/B7 costimulation.

From detailed experiments performed primarily with mice made transgenic for the TCR, it is now clear that the interaction of the TCR expressed on developing immature thymocytes with self peptide-MHC molecules expressed on thymic stromal cells is essential for the selection of those cells that ultimately are destined to leave the thymus and populate the periphery [163-166]. Thus, after productive rearrangement and expression of the αβ chains of the TCR, four fates are possible. If there is little or no affinity of the TCR for self peptide-MHC molecules, the T cells undergo apoptosis as a result of a lack of signal generation. However, if there is a "weak interaction" between the TCR and nonagonist or antagonist self peptide-MHC molecules (i.e. those molecules that have a rapid off-rate from binding to the TCR), the maturing T cells are "positively selected" to survive. Although it still remains controversial, most data are consistent with the notion that an intermediate strength of signal leads to the differentiation of T-Regs. By comparison, if the αβ TCR encounters an agonistic self peptide-MHC interaction, i.e. one that has a slower off-rate and a higher affinity, "negative selection" occurs and these T cells are induced to undergo apoptosis. Consequently, only those T cells that have "nonagonistic" reactivity with self peptide-MHC molecules make up the T cell repertoire.

With regard to the generation of quantal cellular responses, it is noteworthy that IL2 has now been implicated to be involved in both positive and negative selection, as well as T-Reg differentiation.

Recent experiments focused on the signals generated in thymocytes leading to positive selection have revealed that signaling via calcium and calcineurin is necessary for positive selection but dispensable for negative selection[167]. For example, deletion of the regulatory subunit-B1 of calineurin in thymocytes leads to loss of activation of NF-ATc proteins and also inefficient ERK activation, but normal activation of NF-κB/Rel. Of interest, positive selection was found to be markedly deficient in these animals, but negative selection remained intact.

As already discussed, experiments performed with IL2, IL2R, JAK3, and STAT5 (-/-) mice have all now demonstrated that the IL2/IL2R interaction is unnecessary for positive selection. In the absence of signals generated via the IL2/IL2R interaction, positive selection proceeds unimpeded, so that during development and after birth, a normal number and composition of cells mature in the thymus and populate the peripheral lymphoid tissues. Thus, if the "weak" signals between the TCR and self peptide-MHC are not strong enough to trigger expression of the IL2 and IL2R genes, the cells are permitted to survive, and leave the thymus to populate the secondary lymphoid tissues. Should agonistic nonself-peptide-MHC complexes be introduced that are capable of binding to the TCR with high affinity, these T cells, which are non-reactive with "nonagonistic" self peptide-MHC complexes, are not anergic. Rather, if stimulated by non-self agonistic peptide, they are fully capable of expressing IL2 and IL2R genes, and of undergoing IL2-dependent proliferative expansion and differentiation to effector cells in the periphery.

However, the development of T-Regs (i.e. CD4+CD25+ cells) is dependent on the IL2/IL2R interaction. In the absence of IL2 or functional IL2Rs, T-Regs are low or absent, both in the thymus and in the periphery. Moreover, if the IL2/IL2R interaction is restored, either genetically or pharmacologically, then T-Regs are reconstituted and the autoimmune phenomena are delayed or prevented altogether[106,111,168-170]. As well, the function of T-Regs in the periphery is also totally dependent on IL2/IL2R signaling, so that if potential positively selected autoreactive T cells are not continuously suppressed by T-Regs, the IL2 (-/-) syndrome of lymphoid hyperplasia and autoimmunity will occur.

At this juncture, it is logical to propose that the number of triggered TCRs and IL2Rs receptors necessary to generate T-Regs must be higher than the number required to generate simple "positive selection, in that IL2/IL2R gene expression must be triggered[73,171]. In this regard, it has been reported that α chain allelic "inclusion" results in a lower density of TCRs/cell, in that the β chains are paired with 2 distinct α chains in ~ 30% of αβ TCRs[172]. Upon introduction of antigenic peptide, these TCR bi-allelic cells can escape negative selection, presumably because their epitope-specific TCR density is only half normal, and accordingly they would not accumulate the same number of triggered TCRs as a mono-allelic cell. This difference could be responsible for the generation of T-Regs.

It also appears that differentiative signals triggered by the IL2/IL2R interaction are necessary to promote the differentiation to an anergic and suppressive T-Reg cell[81]. Furthermore, the number of triggered TCR receptors must be lower than those necessary to trigger "negative selection". These findings have led some investigators to propose that the main "nonredundant" function of IL2 is to promote the development and function of T-Regs[173,174].

"Negative selection" of potential self-reactive T cells has been proposed to occur via a TCR-triggered apoptosis. The exact molecular mechanisms responsible for this effect still remain obscure, but apoptosis appears to occur when there is a strong agonistic TCR-self-peptide-MHC interaction, which should trigger maximal IL2 and IL2R gene expression. However, data have been reported indicating that negative selection of CD8+ T cells proceeds normally without IL2[175]. By comparison, it remains controversial whether the IL2/IL2R interaction is necessary for negative selection of CD4+ T cells[176].

Quite convincing data have revealed a role for IL2 in CD4+ T cell negative selection [177] using nontransgenic and transgenic IL2-sufficient and deficient animal model systems. It could be shown that during TCR-mediated thymocyte apoptosis, IL2 protein is expressed and detectable *in situ *in the thymus, and apoptotic thymocytes up-regulate the expression of IL2Rs. Furthermore, IL2R+ CD4CD8 double-positive and CD4 single-positive thymocytes undergoing apoptosis bind and internalize IL2. As well, IL2-deficient thymocytes are resistant to TCR/CD3-mediated apoptotic death, which is overcome by providing exogenous IL2 to IL2 (-/-) mice. Finally, disruption or blockade of IL2/IL2R interactions *in vivo *during antigen-mediated negative selection rescues MHC class II restricted thymocytes from apoptosis. Thus, all of these findings provide evidence for the direct involvement of the IL2/IL2R signaling pathway in the deletion of self-reactive double-positive and CD4 single-positive T cells[177].

Accordingly, these data are all entirely consistent with the notion that the CD4+ T cell hyperplasia and autoimmunity observed in IL2 (-/-), IL2R (-/-), and IL2 signaling (-/-) mice are attributable, at least in part, to inefficient deletion of strongly agonistic self-reactive CD4+ T cells, as well as deficient maturation of T-Regs.

## How Can the Immune System Ever Discriminate Between "Self & Non-Self" Peptides?

**Figure 6:**
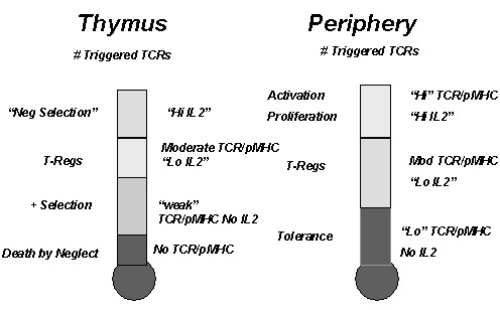
The number of triggered TCRs and IL2Rs determine quantal T cell fates in both the thymus and the periphery. On each plot, the number of triggered TCRs and IL2Rs increase from bottom to top. The different quantal cell fates are dictated by a definite number of triggered Rs as depicted.

The foregoing considerations lead one to the realization that there are no known molecular mechanisms that can explain how the TCR can discriminate *qualitatively *between peptides of self-origin vs. peptides of nonself-origin. Both of these ligands are identical in structure, i.e. they are both peptides. Moreover, the αβ TCRs are also identical structures, whether they recognize self or non-self peptides bound to MHC. It follows that all of the data and logic support a *quantitative *mechanism of discrimination based upon the accumulated number of triggered TCRs and IL2Rs, as shown in Figure 6. Moreover, each triggered cellular differentiative fate of survival, death, anergy, or proliferative expansion, is quantal.

Both in the thymus and in the periphery, there are 3 cellular fates specified by an increasing number of triggered TCRs, which dictates whether IL2 is produced and how much IL2 is produced. Thus, ultimately, the number of IL2-triggered IL2Rs determines the critical quantal fate decisions. A similar conclusion was introduced recently, with the difference that TCR avidity (i.e TCR affinity × density) was postulated to dictate the cell fates, but no role was postulated for IL2[178]. Since ultimately, self-nonself discrimination of the immune system depends on proliferative expansion of antigen-selected clones, the connection between the number of triggered TCRs and IL2Rs offers a molecular explanation for the quantal cellular response.

If T cells that have potential reactivity with self peptide-MHC ligands exit the thymus having escaped negative selection, these T cells will populate the secondary lymphoid tissues in the periphery at very low frequencies, ~ 1 in a million lymphocytes. Thus, these cells make up the TCR repertoire in the periphery, and as long as the distinct self-pMHC complexes remain below the critical number necessary for the formation of a stable synapse, and the TCR-pMHC off-rate is rapid, there is no necessity to introduce any additional mechanism to allow the immune system to ignore self. Instead, in the periphery the immune system only recognizes peptides, whether self or non-self, which are present at a high enough concentration to attain the critical density of 10 peptide-MHC molecules/synapse and as well, that generate a slow enough off-rate from the TCR to form a stable synapse, so that the critical number of triggered TCR/CD28 receptors for activation is reached. The only caveat beyond these considerations is that if an abortive pMHC/TCR synapse forms, the cell receives signals that it interprets as instructions to become anergic, or possibly to differentiate to become suppressive cells (T-Regs), thereby solidifying the non-reactivity on the part of the host to these peptides.

## Conclusions

The "Quantal Theory" states that the fundamental decisions of the T cell immune system are dependent upon the cells receiving a critical number of triggered TCRs and IL2Rs and that the cells respond in an all-or-none fashion. Any reductionist approach to understand how the immune system discriminates self from non-self must begin with a systemic immunological response that most closely correlates with immunity. Thus, the "Quantal Theory" is based on Burnet's axiom that the proliferative expansion of antigen-selected clones is central to the generation of a protective immune response[1]. Secondly, a successful reductionist theory must explain how individual cells of the immune system make the decision to proliferate or not. As each decision of the individual cell is quantal, one must explain the molecular basis of the quantal cellular decision. At each decision point, the assembly of essentially irreversible multicomponent macromolecular complexes underlies the quantal cellular responses. Given the data that the simple IL2/IL2R interaction promotes the quantal decision to undergo cell cycle progression by reaching a critical number of triggered IL2Rs[26], it follows that the quantal decision to express the IL2 gene and the IL2R genes is similarly regulated by a critical number of triggered TCR/CD28 molecules. Thus, the TCR/CD28-triggered expression of the IL2 and IL2R genes is pivotal for the quantal cellular decisions in the thymus that determine distinct fates such as positive selection (survival), the differentiation to T-Regs (anergy), and negative selection (apoptosis), while it is also pivotal for the quantal cellular decisions in the periphery that determine whether to remain unresponsive (survival), to differentiate to T-Regs (anergy), or to begin proliferating (immunity). Accordingly, the "Quantal Theory" offers a unifying explanation at the molecular level that provides the cellular mechanisms for the immune system as a whole to make the quantal discrimination between self antigens and nonself antigens.

These considerations lead to the speculation that non-self peptides that are introduced in low enough concentrations may well be perceived by the immune system as "self" and will generate tolerance. Thus, we now have a molecular, cellular and immunological explanation for the phenomenon of "Low Zone Tolerance", first demonstrated by Mitchison 40 years ago[16]. Accordingly, one might have a means to tolerize individuals to specific defined peptides that may be useful in the treatment of allergy, autoimmunity, and allograft rejection. In contrast, a similar situation could be operative in the tumor-bearing host, and in the host infected chronically with viruses such as the Human Immunodeficiency Virus, and Hepatitis C Virus. In these instances, low persistent antigen levels may well serve to maintain a state of "low zone" tolerance. Accordingly, the question arises as how to break this tolerant state?

## Competing interests

The author declares that he has no competing interests.
